# Transcriptomic and Network Analysis Highlight the Association of Diabetes at Different Stages of Alzheimer’s Disease

**DOI:** 10.3389/fnins.2019.01273

**Published:** 2019-11-29

**Authors:** Jose A. Santiago, Virginie Bottero, Judith A. Potashkin

**Affiliations:** ^1^NeuroHub Analytics, LLC, Chicago, IL, United States; ^2^Department of Cellular and Molecular Pharmacology, The Chicago Medical School, Rosalind Franklin University of Medicine and Science, North Chicago, IL, United States

**Keywords:** Alzheimer’s disease, blood transcriptome, mild cognitive impairment, network analysis, type 2 diabetes

## Abstract

Alzheimer’s disease (AD) and type 2 diabetes (T2D) are among the most prevalent chronic diseases affecting the aging population. Extensive research evidence indicates that T2D is a well-established risk factor for AD; however, the molecular mechanisms underlying this association have not been fully elucidated. Furthermore, how T2D may contribute to the progression of AD is a subject of extensive investigation. In this study, we compared the blood transcriptome of patients with mild cognitive impairment (MCI), AD, and advanced AD to those afflicted with T2D to unveil shared and unique pathways and potential therapeutic targets. Blood transcriptomic analyses revealed a positive correlation between gene expression profiles of MCI, AD, and T2D in seven independent microarrays. Interestingly, gene expression profiles from women with advanced AD correlated negatively with T2D, suggesting sex-specific differences in T2D as a risk factor for AD. Network and pathway analysis revealed that shared molecular networks between MCI and T2D were predominantly enriched in inflammation and infectious diseases whereas those networks shared between overt AD and T2D were involved in the phosphatidylinositol 3-kinase and protein kinase B/Akt (PI3K-AKT) signaling pathway, a major mediator of insulin signaling in the body. The PI3K-AKT signaling pathway became more significantly dysregulated in the advanced AD and T2D shared network. Furthermore, endocrine resistance and atherosclerosis pathways emerged as dysregulated pathways in the advanced AD and T2D shared network. Interestingly, network analysis of shared differentially expressed genes between children with T2D and MCI subjects identified forkhead box O3 (FOXO3) as a central transcriptional regulator, suggesting that it may be a potential therapeutic target for early intervention in AD. Collectively, these results suggest that T2D may be implicated at different stages of AD through different molecular pathways disrupted during the preclinical phase of AD and more advanced stages of the disease.

## Introduction

Alzheimer’s disease (AD) and type 2 diabetes (T2D) are both devastating conditions reaching epidemic numbers worldwide. According to the World Health Organization (WHO), 50 million people worldwide have dementia and approximately 60–70% of the cases are attributed to AD. Accumulation of amyloid β plaques and protein tau in the form of neurofibrillary tangles is a pathological hallmark of the disease. Mutations in the amyloid precursor protein (*APP*), presenilin 1 (*PSEN1*), and presenilin 2 (*PSEN2*) trigger the accumulation of amyloid β plaques and cause early-onset AD (De Strooper and Karran, 2016). Nonetheless, most of the cases present as late onset and are considered sporadic. AD has a long preclinical phase where the disease may start as early as 20 years before the appearance of symptoms (De Strooper and Karran, 2016). This pre-clinical phase is characterized by mild cognitive impairment (MCI) that represents an intermediate stage between the expected cognitive decline occurring during normal aging and dementia.

Likewise, T2D is the most prevalent metabolic disorder affecting over 380 million people worldwide. T2D is characterized by hyperglycemia and the progressive destruction of pancreatic islet β cells, resulting in decreased production of insulin leading to insulin resistance. Increasing evidence from epidemiological studies indicates that T2D is associated with an increased risk of developing AD in several populations (Yang and Song, 2013). Indeed, the presence of T2D results in a two- to threefold higher risk of developing dementia (Cole et al., 2007). Although the exact mechanisms that explain the linkage between T2D and AD are not fully understood, several studies have revealed potential mechanisms underlying this comorbidity. In this regard, impaired insulin signaling and glucose metabolism have been extensively documented as pivotal in the development of dementia and neurodegeneration among T2D patients. For example, insulin resistance has been found to increase the risk of AD (Holscher, 2014). In addition, desensitization of insulin receptors in the brain has been found in both T2D and AD and is suggested to be an early triggering factor in neurodegeneration (Holscher, 2011). Systemic inflammation is also central in the pathogenesis of both T2D and AD. Accumulation of fibrillar proteins in different organs, known as amyloidosis, is a pathological feature of both AD and T2D (Chiti and Dobson, 2006). Deposits of amylin polypeptide in pancreatic islets are present in 95% of T2D patients and it has been demonstrated to impair islet function (Cooper et al., 1987). In fact, both amyloid β and amylin accumulate in tissues in response to infectious agents (Miklossy and McGeer, 2016). Because of the many similar pathogenic mechanisms between AD and T2D, AD has been considered a “type 3 diabetes” by many investigators (de la Monte and Wands, 2008; Zhao et al., 2017).

System biology approaches have significantly expanded our understanding of the molecular pathways disrupted in AD and other neurodegenerative diseases (Santiago et al., 2017b). In particular, network-based approaches integrating transcriptomic data with protein–protein interaction networks have been useful in identifying biomarkers, therapeutic targets, and mechanisms of disease (Santiago and Potashkin, 2014a). In the context of comorbid diseases, network biology has been instrumental in unraveling shared and unique biological pathways (Santiago and Potashkin, 2013, 2014b; Santiago et al., 2016, 2017a). For instance, a network approach using transcriptomic data of post-mortem AD and T2D human brains identified autophagy as the central dysregulated pathway linking both diseases (Caberlotto et al., 2019). Another study constructed AD and T2D networks using knowledge extracted from the scientific literature and revealed a potential interaction between the insulin signaling pathway with the neurotrophin, phosphatidylinositol 3-kinase and protein kinase B/Akt (PI3K-AKT), mammalian/mechanistic target of rapamycin (MTOR), and mitogen-activated protein kinase (MAPK) signaling pathways (Karki et al., 2017). Microarray meta-analysis and pathway analysis identified several pathways, including ephrin receptor, liver X receptor, retinoid X receptor, interleukin 6, and insulin like growth factor 1 as shared between AD and T2D (Mirza et al., 2014).

While these studies provide evidence of shared biological pathways between T2D and AD, they do not explain how T2D may be implicated in the different clinical stages of AD. To this end, we performed a transcriptomic and network analysis of gene expression datasets from MCI, AD, advanced AD, and T2D patients to better understand the shared molecular networks disrupted through the different clinical stages of AD. Here, we reveal unique and common dysregulated pathways between T2D and AD and identify several pathways that are altered during the clinical course of AD, from MCI to more advanced stages of the disease.

## Materials and Methods

### Analysis of Blood Transcriptomic Studies

We used the curated database BaseSpace Correlation Engine (BSCE, Illumina, Inc., San Diego, CA, United States) to search for gene expression studies in MCI, AD, advanced AD, and T2D. Using the search terms “AD,” “MCI,” “diabetes,” “blood,” “human,” “RNA,” and “microarray,” we identified nine studies in blood of MCI, AD, advanced AD, and T2D patients. Two studies on AD patients were removed because they contained less than five samples. Only human microarray studies with five samples or more for cases and controls and curated in BSCE were considered for further analysis. Seven microarrays met our inclusion criteria as of July 01, 2019. Description of microarray datasets included in this study is provided in Table 1.

**TABLE 1:** Blood transcriptomics studies selected for the analysis.

**Datasets**	**Disease**	**Cases**	**Controls**	**Platform**	**PMID**
^∗^GSE63063	Mild cognitive impairment	80	132	Illumina, GPL6947	26343147
GSE63060					23042217
GSE63061					22466004
GSE63063	Alzheimer’s disease	142	132	Illumina, GPL6947	26343147
GSE63060					23042217
GSE63061					22466004
GSE97760	Alzheimer’s disease	9	10	Agilent, GPL16699	25079797
GSE9006	Diabetes	12	24	Affymetrix, Human HG-U133	17595242
GSE13015	Diabetes	5	29	Illumina, GPL6947	19903332
GSE15932	Diabetes	8	8	Affymetrix, Human HG-U133 Plus 2.0	28639886
GSE34198	Diabetes	14	31	Illumina, Human-6 v2.0	29049183
GSE69528	Diabetes	27	28	Illumina, HumanHT-12 V4.0	Not published

*^∗^GSE63063 dataset is composed of two studies: GSE63060 and GSE63061.*

For the studies selected, informed consent was obtained for all subjects according to the Declaration of Helsinki and study protocols were approved by the relevant ethical committees at each clinical site. The study involving AD and MCI subjects is a three-series microarray (GSE63063, GSE63060, and GSE63061) that contains blood transcriptomic data from 142 AD, 80 MCI, and 132 healthy controls from the AddNeuroMed cohort. The AddNeuroMed study is a multi-cohort involving six clinical sites across Europe (Lunnon et al., 2012, 2013; Sood et al., 2015). Diagnosis of AD subjects in the AddNeuroMed cohort was performed according to the National Institute of Neurological and Communicative Disease and Stroke and AD (NINCDS-ADRDA) and Diagnostic and Statistical Manual of Mental Disorders (DSM-IV) (Lunnon et al., 2012, 2013; Sood et al., 2015). Subjects with MCI reported problems with memory, corroborated by an informant, but had normal activities of daily living as specified in the Petersen’s criteria for amnestic MCI. MCI subjects scored 0.5 on the total clinical dementia rating scale (CDR) or had a memory score of 0.5 or 1. All subjects underwent a structured interview and a battery of neuropsychological assessments including the mini mental state examination (MMSE), global deterioration scale (GDS), and CDR by trained researchers. Healthy controls and MCI subjects were further assessed using the CERAD battery. MCI cohort was composed largely of subjects with a likely AD endpoint. Subjects were excluded from the study if they were younger than 65 years, had significant neurological or psychiatric illness other than AD, significant systematic illness or organ failure, or a geriatric depression rating scale score ≥4/5. None of the subjects in the AD, MCI, or control groups had T2D or vascular dementia. More details have been published elsewhere (Lunnon et al., 2012, 2013; Sood et al., 2015). Subjects in the dataset GSE97760 were all female, including patients with advanced AD (*n* = 9, age 79.3 ± 12.3 years) and age-matched female healthy controls (*n* = 10, age 72.1 ± 13.1 years) (Naughton et al., 2015). The AD diagnoses were made by the Neurobehavior and Memory Disorders Clinic at the Ohio State University Wexner Medical Center (NMDC-OSUWMC), following the revised NIH Diagnostic Guidelines for Alzheimer’s disease and Related Disorders (Naughton et al., 2015). All recruited AD subjects were nursing home residents and were completely dependent or bed-ridden, with severe clinical dementia rating 2–3 at the time of recruitment. Healthy controls were recruited among female spouses and primary caregivers of afflicted male dementia patients seen at MDC-OSUWMC. Healthy subjects did not suffer from dementia, acute or chronic infection, inflammation, or diabetes. More details can be found in Naughton et al. (2015).

For the T2D studies, patients were diagnosed with T2D based on criteria of the American Diabetes Association and WHO (Kaizer et al., 2007). In the GSE9006, T2D patients were required to have hemoglobin A1c (HbA1c) levels of 8% or greater. Patients were excluded from the study if they had an active or presumed infection, had other autoimmune disease, were pregnant, were taking immune modulators, or had an initial hematocrit less than 27%. In addition, participants were excluded from the study if they had an active or presumed infection, had other autoimmune disease, were pregnant, or were taking immune modulators (Kaizer et al., 2007). None of the subjects in the T2D or control group had AD or vascular dementia. The information about the diagnosis of T2D in the other studies is not available (GSE13015, GSE15932, GSE34198, and GSE69528).

The genetic overlap among the different gene expression datasets was analyzed for every two datasets. For example, the genetic overlap between the gene expression profiles of MCI individuals and each dataset from T2D was analyzed in BSCE (Kupershmidt et al., 2010). BSCE computes the overlapping *p* values between different gene expression datasets using a “Running Fisher” algorithm described in Kupershmidt et al. (2010). A *p* value of 0.05 or less was considered significant. Microarray meta-analyses were performed in BSCE as described previously (Santiago et al., 2016; Santiago and Potashkin, 2017). The Venn diagrams and the correlation graphs were created using BSCE. For differential gene expression and meta-analysis, differentially expressed genes were extracted from BSCE, and negative values, if any, were replaced with the smallest positive number in the dataset. Genes whose mean normalized test and control intensities were both less than the 20th percentile of the combined normalized signal intensities were removed. To circumvent any potential biases introduced by the use of different array platforms, the meta-analysis tool in BSCE uses a normalized ranking approach, which enables comparability across different gene expression datasets and platforms, independently of the absolute values of fold changes. The scoring and ranking of a gene are calculated based on the activity of the gene in each dataset and the number of datasets in which the gene is differentially expressed. Ranks are normalized to eliminate any bias owing to varying platform and sample size (Kupershmidt et al., 2010). We performed an integrative meta-analysis in which the following conditions had to be met: Firstly, for the array to enter the meta-analysis, it had to show a significant genetic overlap established by the correlation analysis in BSCE software. Secondly, only the group of shared differentially expressed genes in condition A (i.e., MCI, AD, or Advanced AD) and condition B (i.e., T2D) were analyzed further. Specifically, only shared differentially expressed genes in condition A (i.e., MCI, AD, or Advanced AD) and in at least three out of the five T2D studies were included for further network and pathway analysis. Thus, only shared differentially expressed genes between two conditions were analyzed in network and pathway analyses. Only genes with a *p* value of 0.05 or less and an absolute fold-change of 1.2 or greater were included in the analysis.

### Network and Pathway Analysis

Entrez gene identifiers from the genes identified in the meta-analyses were imported into NetworkAnalyst for network and pathway analyses (Xia et al., 2015). In NetworkAnalyst, we used the tissue-specific networks derived from the protein-protein interaction database from human whole blood. Tissue specific data are obtained from DifferentialNet, a database that provides users with differential interactome analysis of human tissues^1^ (Basha et al., 2018). The minimum connected network was selected for further pathway analysis. Pathway analysis data in NetworkAnalyst is derived from the Kyoto encyclopedia of genes and genome (KEGG) and Reactome. For the transcription factor analysis, we used the transcription factor and gene target data derived from the ENCODE ChIP-seq data. Transcription factor analysis uses the BETA Minus Algorithm in which only peak intensity signal <500 and the predicted regulatory potential score <1 is used. Transcription factors were ranked according to network topology measurements including degree and betweenness centrality. The transcription factors Venn diagram was created using the website http://bioinformatics.psb.ugent.be/webtools/Venn/.

## Results

### Correlation of Gene Expression Datasets Between MCI, AD, Advanced AD, and T2D

**FIGURE 1:**
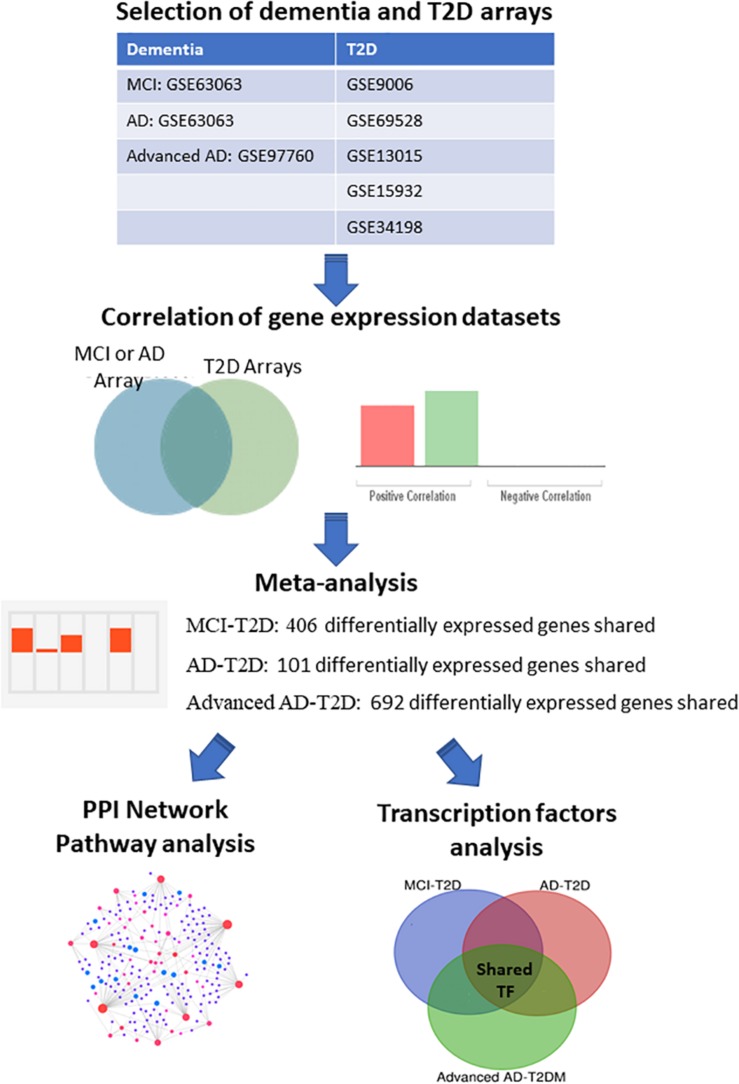
Integrative transcriptomic and network analysis. Overall strategy for the analysis of blood transcriptomic studies from MCI, AD, advanced AD, and T2D patients. Correlation analysis was performed using the bioinformatic platform BSCE. Network and pathway analysis were performed using NetworkAnalyst.

**FIGURE 2:**
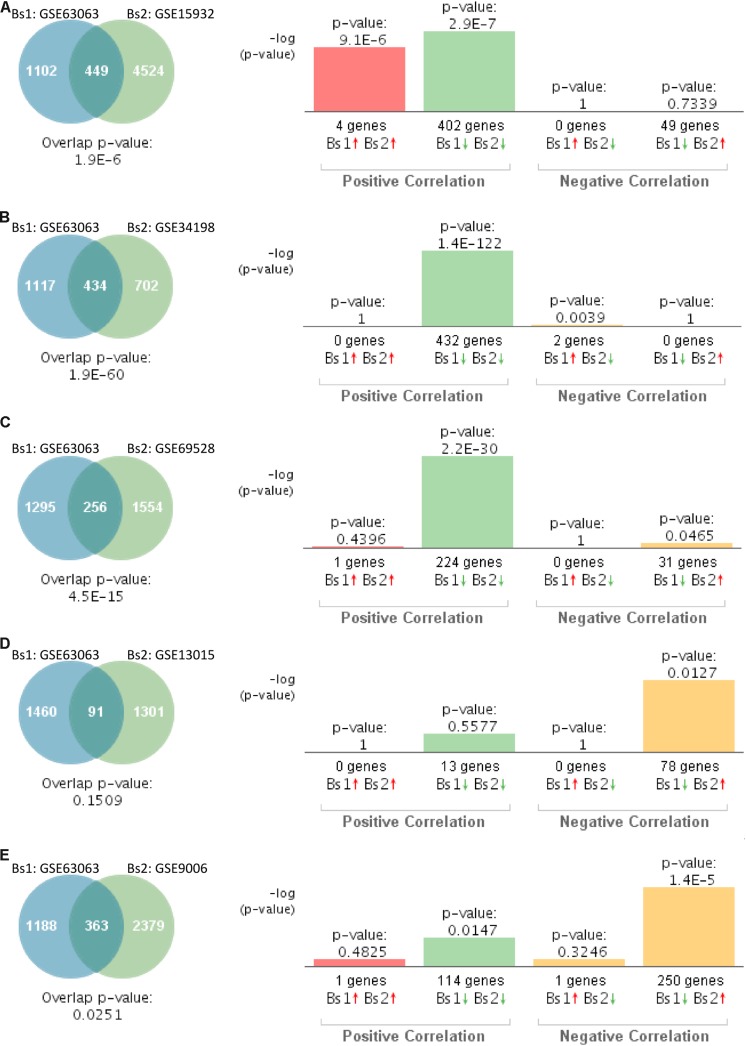
Statistical comparison of the genetic overlap and correlations between subjects with mild cognitive impairment (MCI) and diabetes. **(A)** Venn diagram analysis of shared genes between bioset1 (Bs1) GSE63063 from subjects with MCI and bioset2 (Bs2) GSE15932 from subjects with type 2 diabetes (T2D). Vertical bars represent the significance of the overlap and the correlations between biosets. **(B)** Venn diagram analysis of shared genes between Bs1 GSE63063 from subjects with MCI and Bs2 GSE34198 from subjects with T2D. **(C)** Venn diagram analysis of shared genes between Bs1 GSE63063 from subjects with MCI and Bs2 GSE69528 from subjects with T2D. **(D)** Venn diagram analysis of shared genes between Bs1 GSE63063 from subjects with MCI and Bs2 GSE13015 from subjects with T2D. **(E)** Venn diagram analysis of shared genes between Bs1 GSE63063 from subjects with MCI and Bs2 GSE9006 from subjects with T2D. Red and green arrows denote up- and downregulation, respectively. *p* value is expressed as the –log 10 of the *p* value. Statistical significances regarding the genetic overlap and the directionality of the gene expression changes were derived from the non-parametric ranking method provided by the bioinformatics platform BSCE.

In order to compare the gene expression patterns of individuals with dementia-related conditions to those with T2D, we performed a correlation analysis using BSCE. The overall analysis strategy is presented in Figure 1. Between any two datasets, the numbers of shared differentially expressed genes as well as the directionality of the fold changes were compared. We first investigated the association between MCI and T2D. Gene expression profiles from blood of MCI patients (GSE63063) significantly overlapped with those from individuals with T2D (GSE9006, GSE13015, GSE15932, GSE34198, and GSE69528) in four out of five datasets (Figures 2A–C,E). Correlation analysis indicated that gene expression changes in blood of MCI patients correlated positively with those with T2D in three out of five studies (Figures 2A–C). One study performed on children with T2D (GSE9006) showed a negative correlation with MCI (Figure 2E). In addition, GSE13015 correlated negatively with GSE63063 (*p* = 0.0127), but the overall genetic overlap between the two studies did not reach statistical significance (*p* = 0.1509). Regarding the directionality of the fold changes, most of the overlapping genes were downregulated in both MCI and T2D across all the datasets (Figure 2).

**FIGURE 3:**
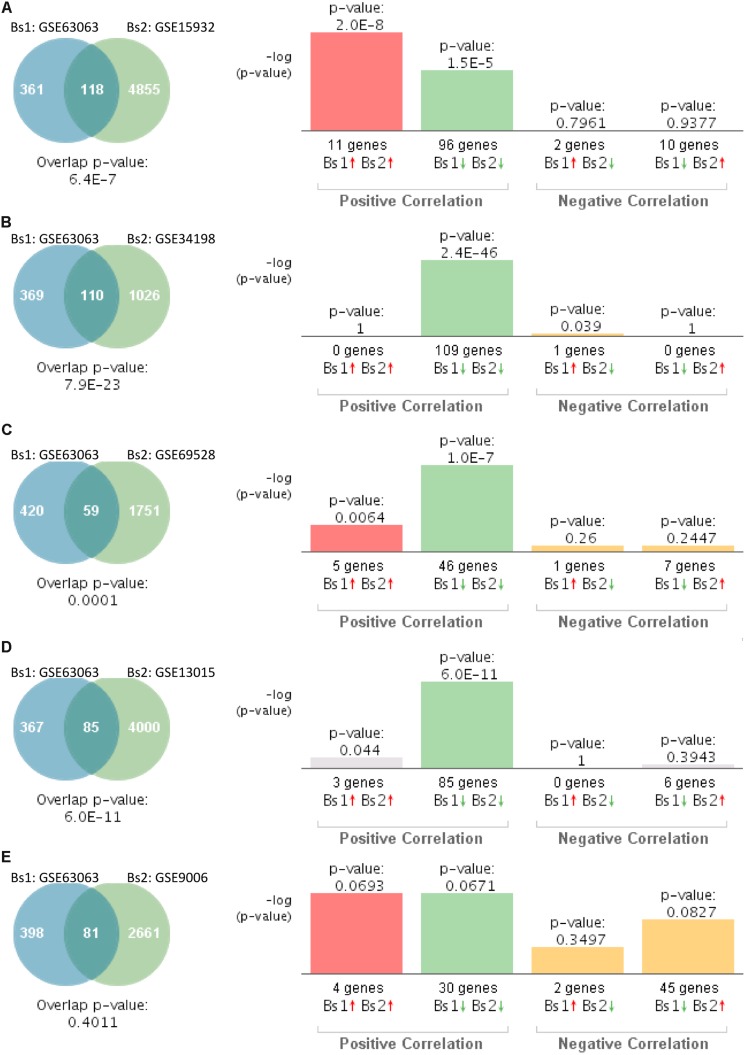
Statistical comparison of the genetic overlap and correlations between subjects with Alzheimer’s disease (AD) and diabetes. **(A)** Venn diagram analysis of shared genes between bioset1 (Bs1) GSE63063 from subjects with AD and bioset2 (Bs2) GSE15932 from subjects with T2D. Vertical bars represent the significance of the overlap and the correlations between biosets. **(B)** Venn diagram analysis of shared genes between Bs1 GSE63063 from subjects with AD and Bs2 GSE34198 from subjects with T2D. **(C)** Venn diagram analysis of shared genes between Bs1 GSE63063 from subjects with AD and Bs2 GSE69528 from subjects with T2D. **(D)** Venn diagram analysis of shared genes between Bs1 GSE63063 from subjects with AD and Bs2 GSE13015 from subjects with T2D. **(E)** Venn diagram analysis of shared genes between Bs1 GSE63063 from subjects with AD and Bs2 GSE9006 from subjects with T2D. Red and green arrows denote up- and downregulation, respectively. *p* value is expressed as the −log 10 of the *p* value. Statistical significances regarding the genetic overlap and the directionality of the gene expression changes were derived from the non-parametric ranking method provided by the bioinformatics platform BSCE.

We next investigated the genetic overlap between AD (GSE63063) and T2D. Gene expression profiles from blood of AD patients significantly overlapped with those individuals with T2D in four out of five datasets (Figures 3A–D). Correlation analysis showed that gene expression patterns in blood of AD patients correlated positively with those affected by T2D in four out of the five studies (Figures 3A–D). The one study performed on children with T2D (GSE9006) showed a positive correlation, but it did not reach significance (Figure 3E, *p* = 0.4011).

**FIGURE 4:**
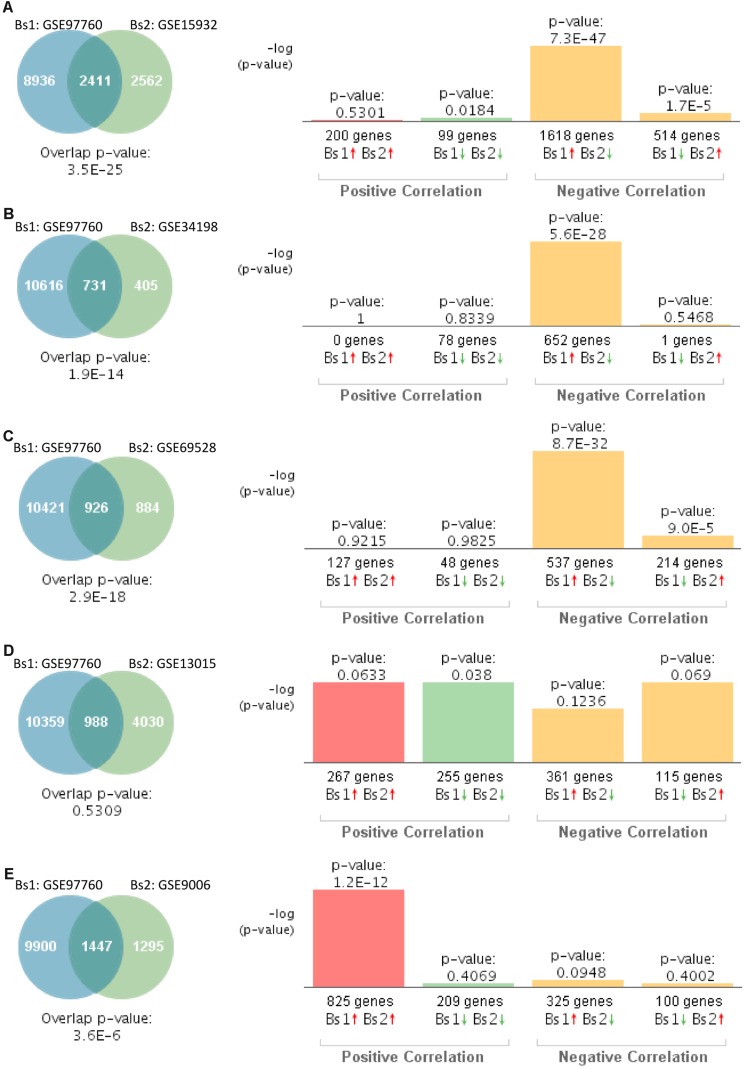
Statistical comparison of the genetic overlap and correlations between subjects with advanced AD and diabetes. **(A)** Venn diagram analysis of shared genes between bioset1 (Bs1) GSE63063 from subjects with advanced AD and bioset2 (Bs2) GSE15932 from subjects with T2D. Vertical bars represent the significance of the overlap and the correlations between biosets. **(B)** Venn diagram analysis of shared genes between Bs1 GSE63063 from subjects with advanced AD and Bs2 GSE34198 from subjects with T2D. **(C)** Venn diagram analysis of shared genes between Bs1 GSE63063 from subjects with advanced AD and Bs2 GSE69528 from subjects with T2D. **(D)** Venn diagram analysis of shared genes between Bs1 GSE63063 from subjects with advanced AD and Bs2 GSE13015 from subjects with T2D. **(E)** Venn diagram analysis of shared genes between Bs1 GSE63063 from subjects with advanced AD and Bs2 GSE9006 from subjects with T2D. Red and green arrows denotes up- and downregulation, respectively. *p* value is expressed as the −log 10 of the *p* value. Statistical significances regarding the genetic overlap and the directionality of the gene expression changes were derived from the non-parametric ranking method provided by the bioinformatics platform BSCE.

Similarly, we compared the genetic overlap between individuals with advanced AD (GSE97760) to those affected by T2D. This particular study of advanced AD was performed on women (Naughton et al., 2015). Gene expression patterns from blood of women with advanced AD significantly overlapped with those individuals with T2D in four out of five studies (Figures 4A–C,E). Correlation analysis indicated that gene expression changes in blood of women with advanced AD correlated negatively with T2D in three out of five studies (Figures 4A–C). The overlap between GSE97760 and GSE13015 was not significant (Figure 4D). The study performed on children with T2D (GSE9006) showed a positive correlation (Figure 4E).

In order to identify potential molecular changes associated with cognitive decline and diabetes occurring at a young age, we compared the blood transcriptome of children with T2D (GSE9006) to those individuals with MCI (GSE63063). A total of 115 differentially expressed genes with the same fold change directionality were shared between children with T2D and MCI individuals (Supplementary Table S1). The transcription factor forkhead box O3 (FOXO3) was the only upregulated gene in both datasets. The remaining 114 differentially expressed genes were downregulated in both T2D and MCI datasets (Supplementary Table S1).

### Integrative Meta-Analysis Between MCI, AD, Advanced AD, and T2D

In order to identify common transcriptional signatures in T2D and AD at different stages, we performed an integrative meta-analysis for each of the following conditions: MCI–T2D, AD–T2D, and advanced AD–T2D. Only datasets with a significant genetic overlap and significant correlations described in the previous section were analyzed in the meta-analysis. Integrative meta-analysis of blood transcriptomic datasets from MCI and T2D individuals resulted in 406 differentially expressed genes shared between the MCI dataset (GSE63063) and at least three out of five T2D studies (Supplementary Table S2). Likewise, meta-analysis of datasets from overt AD and T2D individuals resulted in 101 differentially expressed genes shared between the AD dataset (GSE63063) and at least three out of five T2D studies (Supplementary Table S3). Finally, integrative meta-analysis of datasets from advanced AD and T2D individuals yielded 692 differentially expressed genes shared between the advanced AD dataset (GSE97760) and at least three out of five T2D studies (Supplementary Table S4). The top genes with the highest scores and specificity for each of the meta-analysis are highlighted in the Supplementary Tables S2–S4. The same analyses were repeated excluding the dataset from children with T2D (GSE9006). Integrative meta-analysis from MCI–T2D, AD–T2D, and advanced AD–T2D resulted in 879, 102, and 378 shared differentially expressed genes, respectively. The results from each of the meta-analyses are provided in Supplementary Tables S5–S7.

### Network and Pathway Analysis

**FIGURE 5:**
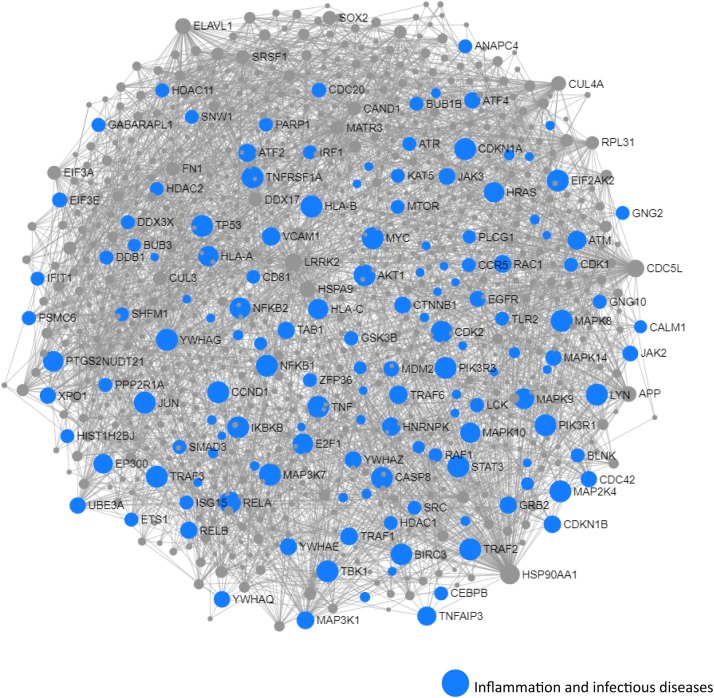
Network and pathway analysis of shared networks between MCI and T2D. Integrative meta-analysis was performed on datasets from MCI and T2D subjects to identify shared dysregulated genes between both diseases. Entrez gene identifiers from the genes identified in the meta-analyses were imported into NetworkAnalyst for network and pathway analyses. In NetworkAnalyst, we selected tissue-specific networks derived from the protein–protein interaction database from human whole blood. The minimum connected network was selected for further pathway analysis. Results from the pathway analysis are derived from the Kyoto encyclopedia of genes and genome (KEGG) and Reactome. Shared networks between MCI and T2D were enriched predominantly in genes associated to infectious diseases and inflammation (blue circles).

**FIGURE 6:**
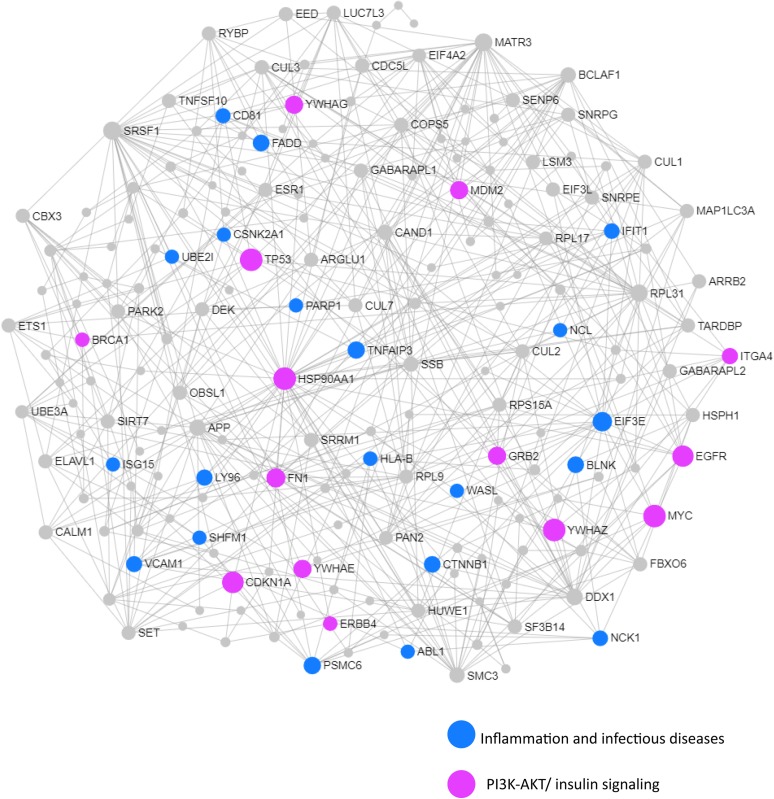
Network and pathway analysis of shared networks between AD and T2D. Integrative meta-analysis was performed on datasets from AD and T2D subjects to identify shared dysregulated genes between both diseases. Entrez gene identifiers from the genes identified in the meta-analyses were imported into NetworkAnalyst for network and pathway analyses. In NetworkAnalyst, we selected tissue-specific networks derived from the protein–protein interaction database from human whole blood. The minimum connected network was selected for further pathway analysis. Results from the pathway analysis are derived from the KEGG and Reactome. Shared networks between AD and T2D were enriched predominantly in genes associated to infectious diseases and inflammation (blue circles) and the phosphatidylinositol 3-kinase and protein kinase B (PI3K-AKT) signaling pathway (pink circles).

**FIGURE 7:**
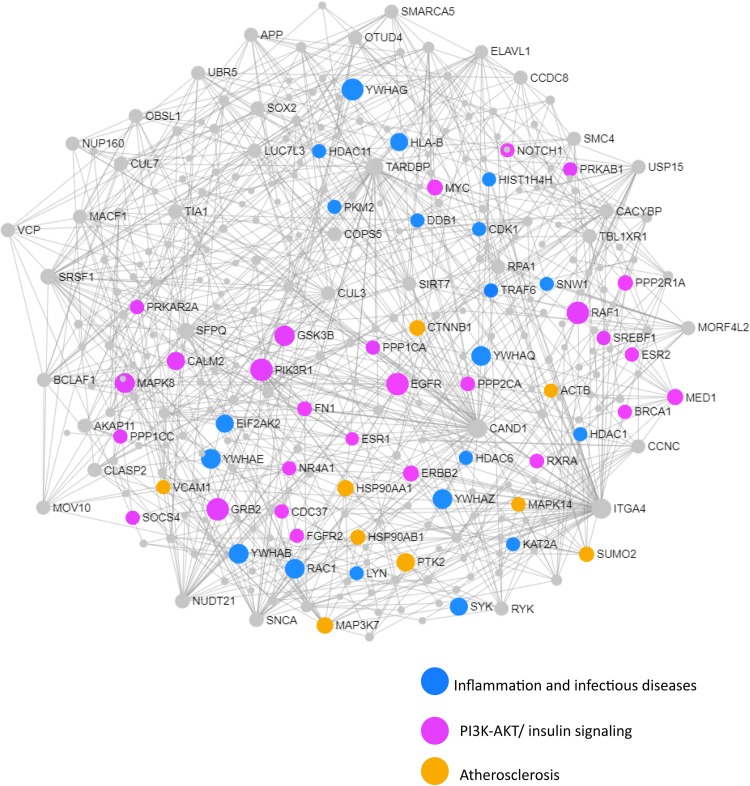
Network and pathway analysis of shared networks between advanced AD and T2D. Integrative meta-analysis was performed on datasets from advanced AD and T2D subjects to identify shared dysregulated genes between both diseases. Entrez gene identifiers from the genes identified in the meta-analyses were imported into NetworkAnalyst for network and pathway analyses. In NetworkAnalyst we selected tissue specific networks derived from the protein-protein interaction database from human whole blood. The minimum connected network was selected for further pathway analysis. Results from the pathway analysis are derived from the KEGG and Reactome. Shared networks between advanced AD and T2D were enriched predominantly in genes associated to infectious diseases and inflammation (blue circles), the phosphatidylinositol 3-kinase and protein kinase B (PI3K-AKT) signaling pathway (pink circles) and atherosclerosis (orange circles).

**FIGURE 8:**
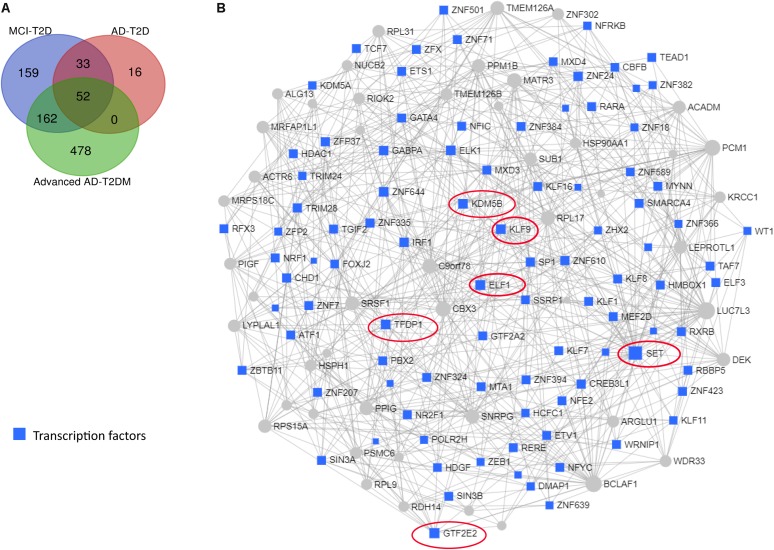
Transcription factor and network analysis. Network and transcription factor analysis was performed using NetworkAnalyst. **(A)** Transcription factor data were derived from the ENCODE ChIP-seq database. **(B)** Transcription factors (blue rectangles) were ranked according to network topology measurements, degree, and betweenness centrality. Gray lines represent protein–protein interactions. Transcription factors with the highest values of degree and betweenness centrality measurements are enclosed in red ovals.

We next performed a network and pathway analysis using the shared genes identified in the meta-analyses. Using NetworkAnalyst (Xia et al., 2015), tissue-specific networks derived from the protein–protein interaction database from human whole blood were constructed for each of the following conditions separately: MCI–T2D, AD–T2D, and advanced AD–T2D. The minimum connected network was selected for further analysis. Network analysis of shared genes between MCI and T2D resulted in a network predominantly enriched in inflammatory pathways and infectious diseases (Figure 5). The most significantly altered pathways were hepatitis B, Epstein–Barr virus infection, human T-cell lymphotropic virus type 1 (HTLV-1) infection, Kaposi’s sarcoma-associated herpesvirus, nuclear factor-kappa B (NFKB) signaling pathway, hepatitis C, and tumor necrosis factor (TNF) signaling pathway. Network and pathway analyses of shared genes between AD and T2D resulted in a network enriched in genes associated with infectious diseases and inflammation, including hepatitis C, *Escherichia coli* infection, Epstein–Barr virus infection, nuclear factor kappa B (NFKB) signaling pathway, and the PI3K-AKT signaling pathway (Figure 6). Lastly, network and pathway analysis of shared genes between advanced AD and T2D resulted in a network predominantly enriched in genes involved in PI3K-AKT signaling, inflammation, and fluid shear stress and atherosclerosis (Figure 7). The ubiquitin-mediated proteolysis pathway was the highest ranked pathway among three group comparisons. The top 20 pathways for each group comparison are listed in Table 2. As noted in Table 2, the PI3K-AKT signaling pathway becomes more significantly dysregulated in the network associated with advanced AD and T2D (*p* = 1.01E-06) compared to the AD–T2D shared network (*p* = 9.40E-04). These results from the pathway analysis were sustained after repeating the meta-analyses excluding the dataset from children with T2D (GSE9006) (Supplementary Table S8). In order to identify key transcriptional regulators of the shared genes identified in the meta-analysis, a transcription factor analysis was performed using NetworkAnalyst. Venn diagram analysis identified 52 genes shared among the three groups: MCI–T2D, AD–T2D, and advanced AD–T2D (Figure 8A). Transcription factor analysis was performed using the 52 shared genes and were ranked according to network topology measurements, including degree and betweenness centrality (Figure 8B). The most highly ranked transcription factors were SET, GTF2E2, ELF1, TFDP1, KDM5B, and KLF9 (Figure 8B).

**TABLE 2:** Pathway and network analysis of shared networks.

**MCI–T2D**		**AD–T2D**		**Advanced AD–T2D**	
**Pathway**	***p* value**	**Pathway**	***p* value**	**Pathway**	***p* value**
Ubiquitin-mediated proteolysis	8.59E-23	Ubiquitin-mediated proteolysis	1.94E–10	Ubiquitin-mediated proteolysis	1.49E-09
Pathways in cancer	5.50E–21	Cell cycle	4.50E–09	Viral carcinogenesis	3.56E–08
Hepatitis B	5.26E–19	Hepatitis C	5.74E–07	Hepatitis C	3.81E–07
Viral carcinogenesis	1.48E–17	Spliceosome	5.51E–06	Cell cycle	5.35E–07
ErbB signaling pathway	2.74E–17	Pathogenic *Escherichia coli* infection	1.78E–05	PI3K-Akt signaling pathway	1.01E–06
Cell cycle	4.44E–17	ErbB signaling pathway	4.17E–05	Endometrial cancer	1.72E–06
Neurotrophin signaling pathway	5.63E–17	Viral carcinogenesis	5.19E–05	Hippo signaling pathway	1.77E–06
Epstein–Barr virus infection	3.51E–16	Pathways in cancer	1.32E–04	Neurotrophin signaling pathway	1.82E–06
HTLV-I infection	8.76E–16	Chronic myeloid leukemia	1.47E–04	Pathways in cancer	3.92E–06
Kaposi’s sarcoma-associated herpesvirus infection	1.20E–15	Epstein–Barr virus infection	2.33E–04	Endocrine resistance	6.68E–06
FoxO signaling pathway	1.85E–14	Endometrial cancer	2.35E–04	Thyroid hormone signaling pathway	7.41E–06
Chronic myeloid leukemia	2.75E–14	Bladder cancer	3.66E–04	Bacterial invasion of epithelial cells	1.66E–05
Colorectal cancer	1.61E–13	Breast cancer	3.89E–04	Protein processing in endoplasmic reticulum	1.93E–05
NF-kappa B signaling pathway	3.82E–13	Mitophagy – animal	4.39E–04	MAPK signaling pathway	1.93E–05
Endometrial cancer	5.88E–13	Renal cell carcinoma	6.07E–04	IL-17 signaling pathway	2.28E–05
Pancreatic cancer	1.13E–12	Prostate cancer	6.664E–04	Prostate cancer	3.40E–05
Hepatitis C	1.16E–12	Endocrine resistance	7.07E–04	Focal adhesion	4.68E–05
Prostate cancer	5.69E–12	NF–kappa B signaling pathway	7.97E–04	Estrogen signaling pathway	4.85E–05
TNF signaling pathway	7.00E–12	FoxO signaling pathway	8.81E–04	Wnt signaling pathway	4.90E–05
Prolactin signaling pathway	9.68E–12	PI3K–Akt signaling pathway	9.36E–04	Fluid shear stress and atherosclerosis	5.23E–05

*Pathway analysis was performed using Network Analyst and data derived from the kyoto encyclopedia of genes and genome (KEGG) and Reactome. MCI, mild cognitive impairment; AD, Alzheimer’s disease; T2D, type 2 diabetes.*

**FIGURE 9:**
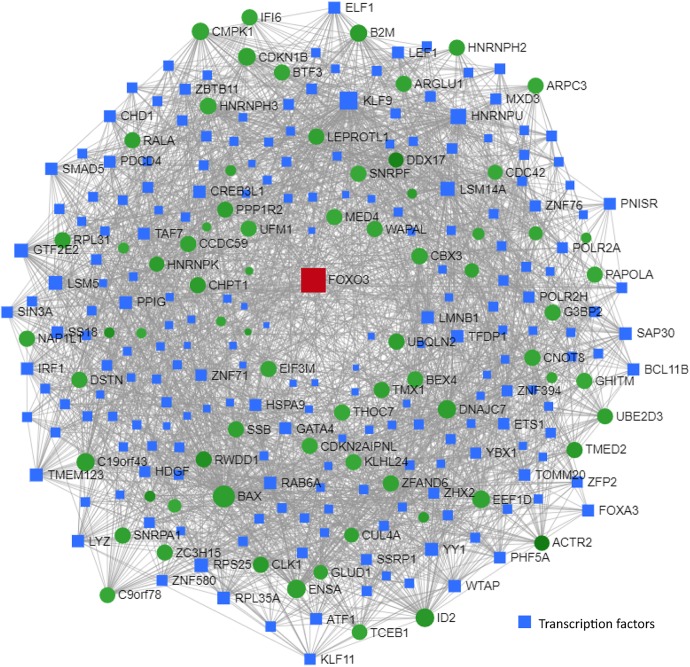
Transcription factor and network analysis. Network and transcription factor analysis was performed using NetworkAnalyst. Transcription factor and network analysis of shared differentially expressed genes between children with T2D (GSE9006) and MCI individuals (GSE63063). Transcription factor data were derived from the ENCODE ChIP-seq database. Transcription factors (blue rectangles) were ranked according to network topology measurements, degree, and betweenness centrality. Gray lines represent protein–protein interactions. Red and green colors denote upregulation and downregulation, respectively.

Network and transcription factor analysis of shared genes between children with T2D and MCI individuals identified FOXO3 as a central transcriptional regulator (Figure 9). This network was enriched in genes associated with TGF-β signaling pathway, thyroid hormone signaling pathway, Huntington’s disease, and longevity regulating pathway.

## Discussion

Mounting evidence from epidemiological and preclinical studies indicates that T2D is a major contributing factor in the pathogenesis of AD. Although evidence has established that AD and T2D share common dysregulated biological pathways, the precise pathophysiological mechanisms underlying this association have not been fully elucidated. Importantly, how T2D may be implicated in the progression from MCI to AD is poorly understood. To this end, we analyzed blood transcriptomic data from MCI, AD, advanced AD, and T2D patients using both meta-analyses and network analyses to investigate the molecular basis for this comorbidity.

We first compared the blood transcriptome of MCI, AD, and advanced AD subjects to those affected by T2D. We found a significant genetic overlap between the dataset from MCI subjects and those datasets from T2D patients. Gene expression profiles from MCI individuals correlated positively with those from T2D patients. Likewise, gene expression profiles from AD subjects had a significant genetic overlap and correlated positively with those datasets from T2D patients. These findings are consistent with the numerous epidemiological studies that have found a positive association between T2D and AD (Biessels et al., 2006; Kopf and Frolich, 2009; Yang and Song, 2013).

Interestingly, gene expression profiles from women with advanced AD had a significant genetic overlap with those from T2D patients; however, contrary to MCI and AD, this dataset correlated negatively with most of the T2D studies. In this regard, sex-related differences in AD and diabetes are the subject of extensive investigation. The prevalence of T2D is higher in men than in women (Wild et al., 2004), and AD is more prevalent in women than in men (Riedel et al., 2016). These differences, however, may be partly explained by the fact that women have a longer life expectancy than men. Although sex differences have been documented for both diseases independently, sex differences in T2D as a risk factor for AD are less understood. In a meta-analysis of 14 studies comprising more than 100,000 cases of dementia, women with T2D had a 19% greater risk of developing vascular dementia, but not AD (Chatterjee et al., 2016). Sex-specific differences in T2D as a risk factor for AD are poorly understood and more research is needed to further clarify these findings. Also, the results presented herein should be taken with caution as they represent correlative values and not causal relationships. A negative correlation between women with AD and T2D, although intriguing, cannot be explained precisely with the datasets currently available. A more rigorous analysis evaluating diabetic women and the risk of AD is needed to better understand this association.

Network and pathway analysis revealed several similarities and differences in dysregulated pathways among the three groups, MCI–T2D, AD–T2D, and advanced AD–T2D. Not surprisingly, inflammatory pathways were central in the MCI–T2D and AD–T2D networks. For example, the MCI–T2D network was enriched in genes associated with infectious diseases and inflammation, including hepatitis B and C, Epstein–Barr virus infection, HTLV-1 infection, Kaposi’s sarcoma-associated herpesvirus, NFKB, and TNF signaling pathways. In this context, inflammation is a central pathogenic mechanism to all neurodegenerative diseases and metabolic disorders including T2D. Increased levels of proinflammatory cytokines are found in AD brains at very early stages of the disease (Wyss-Coray, 2006). Activation of key inflammatory pathways including TNF signaling pathway is found in the cerebrospinal fluid of MCI patients (Pillai et al., 2019). Furthermore, elevated levels of proinflammatory cytokines TNF-alpha and decreased production of the anti-inflammatory cytokines TGF-beta have been documented in patients with MCI at risk of AD (Tarkowski et al., 2003). Numerous studies have documented the involvement of different pathogens in the development of cognitive decline and AD (Sochocka et al., 2017). Viral pathogens including human herpes virus, Epstein–Barr virus, cytomegalovirus, and hepatitis C virus have been linked to cognitive impairment and AD (Hemling et al., 2003; Licastro et al., 2014; Sochocka et al., 2017). Similarly, bacterial infections such as *Chlamydia pneumoniae* and *Helicobacter pylori* have been associated with an increased risk of cognitive decline and AD (Bibi et al., 2014; Miklossy, 2015). In the context of T2D, *H. pylori* infections have been positively associated to T2D in several studies (Demir et al., 2008; Hsieh et al., 2013; Han et al., 2016). Thus, inflammation caused by infectious pathogens is a major shared mechanism between T2D and AD. According to the evidence from our pathway analysis, inflammation is a predominant pathway early in the preclinical phase of AD.

Similarly, the AD–T2D network was enriched in genes related to inflammation and infectious diseases but to a lesser extent. Infectious diseases and inflammatory pathways had a lower significance values compared to those obtained in the MCI–T2D network. In contrast to the MCI–T2D network, we observe the presence of genes related to endocrine resistance and the PI3K-AKT signaling pathways. In this regard, the PI3K-AKT signaling pathway is a key mediator of the insulin effects in the body. Activation of the PI3K-AKT signaling pathway facilitates glucose uptake in peripheral tissues. In the brain, insulin effects and the PI3K-AKT signaling pathway are involved in neuronal health, synaptic plasticity, and neuroprotection (Hui et al., 2005; van der Heide et al., 2005). Furthermore, the PI3K-AKT pathway is associated with the activation and function of microglia, which are important resident cells of the central nervous system responsible for neuroinflammation (Zhong et al., 2017; Xu et al., 2018). More importantly, anti-diabetic drugs have been shown to prevent amyloid beta neurotoxicity in AD models through the activation of the PI3K-AKT pathway (Cai et al., 2014; Liu et al., 2016; Tumminia et al., 2018). Thus, targeting the PI3K-AKT pathway could be a potential therapeutic target for AD.

In the advanced AD–T2D network, we observed that the significance of the PI3K-AKT signaling pathway increased as more genes involved in this pathway became dysregulated. In addition, estrogen signaling and atherosclerosis pathways became dysregulated. This is not surprising since increasing evidence from epidemiological studies suggest a link between AD and atherosclerosis. Several hypotheses have emerged explaining this association. For instance, some evidence suggests that AD may result as a consequence of atherosclerosis of cranial vessels or brain infarctions (Casserly and Topol, 2004). Another explanation is that AD and atherosclerosis are independent but have convergent biological pathways (Casserly and Topol, 2004). For example, elevated serum cholesterol, inflammation, and shared genetic risk factors are some of the potential mechanisms linking atherosclerosis and AD. One of the shared genetic risk factors with the strongest evidence is the ε4 allele of the apolipoprotein E gene (APOE). APOEε4 confers a modest risk for atherosclerosis (Wilson et al., 1996), and it is the strongest genetic risk factor for AD (Farrer et al., 1997). The results from the network and pathway analysis suggest that at advanced stages of AD, the impairment of insulin signaling worsens and the cardiovascular system gets compromised. In this regard, cardiovascular factors have been good predictors of progression and greater decline in AD (Mielke et al., 2007), but there is some conflicting evidence (Javanshiri et al., 2018). Future studies will be focused at investigating the molecular networks and shared pathways between cardiovascular diseases and AD.

Network analysis identified several transcription factors implicated in the pathogenesis of AD. The most highly ranked transcription factor was SET nuclear proto-oncogene (SET), an endogenous inhibitor of the protein phosphatase 2A (PP2A), which is a major tau dephosphorylating enzyme in the brain largely implicated in the pathogenesis of AD (Theendakara et al., 2017). SET has been implicated in the neuronal apoptotic pathway in AD (Wu et al., 2018). Another highly ranked transcription factor was ETS transcription factor 1 (ELF1). Interestingly, the green tea polyphenol epigallocatechin-3-*O*-gallate (EGCG) increases the expression of the toll interacting protein TLR4 by suppressing the expression of ELF1 (Kumazoe et al., 2017). This finding is of great importance given the fact that TLR4 signaling links innate immunity with fatty acid-induced insulin resistance (Shi et al., 2006). In fact, TLR4 is suggested to be a molecular link among nutrition, lipids, inflammation, insulin resistance, and AD (Shi et al., 2006; Huang et al., 2017). Similarly, krüppel-like factor 9 (KLF9) was among the highest-ranked transcription factors. KLF9 promotes the expression of peroxisome proliferator-activated receptor γ coactivator 1α (PGC1α), resulting in hepatic gluconeogenesis, and it is involved in glucocorticoid-induced hyperglycemia and diabetes (Cui et al., 2019; Sweet et al., 2019). Given the involvement of these transcription factors in key processes associated with AD, their potential as therapeutic targets warrants further investigation.

Understanding the molecular networks associated with AD that become disrupted at a young age might shed light on mechanisms leading to cognitive impairment and AD later in life. Several processes associated with the pathogenesis of AD, including the accumulation of neurofibrillary tangles of hyperphosphorylated tau and amyloid plaques have been found decades before disease onset (Seeley et al., 2009; Braak and Del Tredici, 2011; Axelrud et al., 2019). In fact, postmortem brains of 38 out of 42 individuals between the ages of 4 and 29 displayed abnormally phosphorylated tau protein, suggesting that AD-related pathogenic processes may start early before puberty or in early young adulthood (Braak and Del Tredici, 2011). Recently, a functional magnetic resonance study showed an increased connectivity among regions susceptible to tau pathology in children and adolescents with higher susceptibility to AD (Axelrud et al., 2019). Based on these intriguing findings, we investigated what molecular changes in diabetic children could be associated with the development of cognitive decline at a later age. Among the shared genes between T2D and MCI individuals, FOXO3 was the only gene upregulated in both conditions. Network analysis of shared differentially expressed genes between children with T2D and MCI subjects resulted in a network centered on FOXO3. FOXO3 is a master transcriptional regulator implicated in multiple processes, including increased lifespan, healthy aging, gluconeogenesis, autophagy, apoptosis, proteostasis, and breakdown of reactive oxygen species (Morris et al., 2015). Not surprisingly, FOXO3 has been involved in several pathological mechanisms in AD. For example, decreased levels of miR-132 and miR-212 leads to upregulation of FOXO3 and causes apoptosis of primary neurons (Wong et al., 2013). Furthermore, levels of both FOXO3 and its activator P300 are significantly upregulated in the hippocampus of AD patients (Blalock et al., 2004). Consistent with these findings, we found that FOXO3 is significantly upregulated in blood of T2D children and MCI subjects. Strikingly, calorie restriction and subsequent activation of the insulin receptor signaling pathway leads to inactivation of FOXO3 and attenuation of amyloid neuropathology in a mouse model of AD (Qin et al., 2008). This study suggested that a calorie restriction dietary regime may prevent AD through regulation of the PI3K-AKT-FOXO3 signaling pathway. These results suggest that FOXO3 may be a potential therapeutic target for early intervention and underscore the importance of nutrition in AD.

Pathway analysis of shared genes between diabetic children and MCI subjects identified several dysregulated pathways, including TGF-β and thyroid hormone signaling pathways, Huntington’s disease, and longevity regulating pathway. In this regard, impaired TGF-β signaling has been demonstrated to contribute to AD neurodegeneration through several mechanisms including microglial activation, cell-cycle reactivation, and increased levels of secreted Aβ (Das and Golde, 2006; Tesseur et al., 2006). Dysregulation of TGFβ signaling pathway has been shown to be an early event specific to the AD brain, not present in other neurodegenerative diseases (Caraci et al., 2012). In this context, expression of the TGFβ-II receptor is reduced very early in the course of AD and promoted the deposition of toxic Aβ in a mouse model of AD (Tesseur et al., 2006). Thus, targeting TGFβ signaling pathway and FOXO3 transcription factor may be a potential neuroprotective strategy to prevent AD.

Collectively, the results presented in this study suggest that T2D may be associated to cognitive decline and AD through different biological pathways. Early in the preclinical phase of AD, the shared biological pathways between MCI and T2D are mostly related to inflammation and infectious diseases, whereas later as the disease progresses, in AD–T2D, the insulin signaling becomes dysregulated and the cardiovascular system gets compromised. However, these findings need to be taken with caution as they are based on bioinformatic and correlational analyses. In addition, unanticipated confounds including differences in array platforms, blood collection, and RNA extraction methods; methods of ascertainment; and different criteria used to define patient populations may bias the results. Whether T2D is associated with a more rapid progression in AD is still unclear. A more in-depth study using transcriptomic, proteomic, and clinical data taken longitudinally will be crucial to assess the validity of these findings. Equally important will be to investigate how specific dietary patterns impact regulatory networks involved in neuroprotection and neurodegeneration. By interrogating gene expression datasets from AD and T2D patients with those of individuals adhering to a Mediterranean diet, nutritional lifestyle changes that slow or prevent dementia may be revealed.

## Data Availability Statement

All datasets generated for this study are included in the article/Supplementary Material.

## Author Contributions

JS and JP conceived and designed the experiments, and wrote the manuscript. JS performed the experiments. JS, VB, and JP analyzed the data.

## Conflict of Interest

JS is employed by the company NeuroHub Analytics, LLC. The remaining authors declare that the research was conducted in the absence of any commercial or financial relationships that could be construed as a potential conflict of interest.

## Footnotes

1 http://netbio.bgu.ac.il/diffnet/
