# Supplementary material for: Transcriptomic and Network Analysis Highlight the Association of Diabetes at Different Stages of Alzheimer’s Disease
Source: Front Neurosci. 2019 Nov 29;13:1273. doi: 10.3389/fnins.2019.01273 (PMC6895844; doi:10.3389/fnins.2019.01273)
Supplement: Supplementary file 8 [file Table_8.docx]

**Supplementary table S8. Pathway analysis of shared networks excluding the dataset from children with diabetes (GSE9006).**

| **MCI-T2D** | | **AD-T2D** | | **Advanced AD-T2D** | |
| --- | --- | --- | --- | --- | --- |
| **Pathway** | **P value** | **Pathway** | **P value** | **Pathway** | **P value** |
| Epstein-Barr virus infection | 1.06E-37 | Ubiquitin mediated proteolysis | 9.24E-30 | Cell cycle | 8.47E-31 |
| Hepatitis B | 2.43E-28 | Epstein-Barr virus infection | 1.02E-29 | Pathways in cancer | 7.77E-29 |
| Ubiquitin mediated proteolysis | 1.81E-27 | Viral carcinogenesis | 5.56E-29 | Epstein-Barr virus infection | 1.12E-28 |
| Pathways in cancer | 1.39E-26 | Cell cycle | 2.36E-24 | Hepatitis B | 3.43E-28 |
| Viral carcinogenesis | 2.20E-26 | Hepatitis B | 1.06E-22 | Viral carcinogenesis | 2.75E-27 |
| Kaposi's sarcoma-associated herpesvirus infection | 1.86E-22 | Pathways in cancer | 1.13E-21 | Ubiquitin mediated proteolysis | 4.03E-27 |
| Cell cycle | 5.20E-21 | Apoptosis | 6.83E-17 | HTLV-I infection | 1.83E-21 |
| Pancreatic cancer | 1.73E-20 | HTLV-I infection | 1.07E-16 | Pancreatic cancer | 2.05E-19 |
| Apoptosis | 5.31E-20 | Kaposi's sarcoma-associated herpesvirus infection | 2.53E-16 | Kaposi's sarcoma-associated herpesvirus infection | 5.11E-19 |
| FoxO signaling pathway | 6.94E-19 | Hepatitis C | 9.22E-16 | Neurotrophin signaling pathway | 1.51E-18 |
| Neurotrophin signaling pathway | 6.43E-18 | Pancreatic cancer | 1.38E-15 | Apoptosis | 1.96E-18 |
| NF-kappa B signaling pathway | 6.73E-18 | Chronic myeloid leukemia | 2.73E-15 | Chronic myeloid leukemia | 4.86E-18 |
| HTLV-I infection | 7.69E-18 | NF-kappa B signaling pathway | 1.91E-14 | Hepatitis C | 2.43E-17 |
| Measles | 1.85E-17 | Neurotrophin signaling pathway | 8.50E-14 | Thyroid hormone signaling pathway | 1.26E-16 |
| Colorectal cancer | 2.23E-17 | Cellular senescence | 9.56E-14 | TNF signaling pathway | 2.69E-16 |
| Chronic myeloid leukemia | 2.43E-17 | Colorectal cancer | 2.02E-13 | Adherens junction | 3.52E-16 |
| Hepatitis C | 5.50E-17 | Toxoplasmosis | 4.70E-13 | Measles | 5.66E-16 |
| Proteoglycans in cancer | 1.54E-16 | Protein processing in endoplasmic reticulum | 5.69E-13 | FoxO signaling pathway | 5.49E-15 |
| Cellular senescence | 5.42E-16 | Measles | 8.51E-13 | T cell receptor signaling pathway | 9.15E-15 |
| TNF signaling pathway | 6.07E-16 | FoxO signaling pathway | 1.20E-12 | Protein processing in endoplasmic reticulum | 1.54E-14 |
| T cell receptor signaling pathway | 2.19E-15 | Proteoglycans in cancer | 1.51E-12 | ErbB signaling pathway | 6.36E-14 |
| Mitophagy - animal | 4.26E-15 | Insulin signaling pathway | 2.17E-12 | AGE-RAGE signaling pathway in diabetic complications | 1.08E-13 |
| Adherens junction | 1.87E-14 | MAPK signaling pathway | 4.51E-12 | Endocrine resistance | 6.39E-13 |
| Pathogenic Escherichia coli infection | 2.75E-14 | PI3K-Akt signaling pathway | 6.15E-12 | Insulin signaling pathway | 3.67E-12 |
| Shigellosis | 3.10E-14 | Thyroid hormone signaling pathway | 8.54E-11 | Fluid shear stress and atherosclerosis | 2.60E-10 |

**Supplementary table S8.** Pathway analysis of shared networks excluding the dataset from children with diabetes (GSE9006). Pathway analysis was performed using NetworkAnalyst and data derived from the Kyoto Encyclopedia of Genes and Genome (KEGG) and Reactome. MCI=mild cognitive impairment, AD= Alzheimer’s disease, T2D = type 2 diabetes. Blue font denotes pathways related to inflammation and infectious diseases. Pink font denotes pathways related to insulin signaling and endocrine resistance. Orange font denotes pathways related to atherosclerosis.
